# Photodynamic and Antibacterial Assessment of Gold Nanoparticles Mediated by Gold (III) Chloride Trihydrate and Sodium Citrate under Alkaline Conditions

**DOI:** 10.3390/ma17133157

**Published:** 2024-06-27

**Authors:** Chien-Wei Cheng, Shwu-Yuan Lee, Tang-Yu Chen, Ching-Chuan Chen, Hsien-Tsung Tsai, Hsuan-Han Huang, Jeu-Ming P. Yuann, Ji-Yuan Liang

**Affiliations:** 1 Department of Biotechnology, Ming Chuan University, Taoyuan City 33343, Taiwan; ochien@gmail.com (C.-W.C.); leo14101057@gmail.com (T.-Y.C.); 0978652432abc@gmail.com (C.-C.C.); 2 Department of Tourism and Leisure, Hsing Wu University, New Taipei City 24452, Taiwan; 091007@mail.hwu.edu.tw; 3 Tea and Beverage Research Station, Taoyuan City 32654, Taiwan; tsung@tbrs.gov.tw (H.-T.T.); a1009@tbrs.gov.tw (H.-H.H.)

**Keywords:** aPDI, gold chloride, gold nanoparticles, sodium citrate, violet light

## Abstract

Sodium citrate (SC) is sensitive to violet light illumination (VLI) and acts as a weak reductant. Conversely, gold (III) chloride trihydrate (GC) often acts as an oxidant in a redox reaction. In this study, the influences of colored light on the production of gold nanoparticles (AuNPs) in a mixture of gold (III) ions and citrate via VLI and the antibacterial photodynamic inactivation (aPDI) of *Escherichia coli* (*E. coli*) are determined under alkaline conditions. The diameter of AuNPs is within the range of 3–15 nm, i.e., their mean diameter is 9 nm; when citrate is mixed with gold (III) ions under VLI, AuNPs are formed via an electron transfer process. Additionally, GC mixed with SC (GCSC) inhibits *E. coli* more effectively under VLI than it does under blue, green, or red light. GCSC and SC are shown to inhibit *E. coli* populations by 4.67 and 1.12 logs, respectively, via VLI at 10 W/m^2^ for 60 min under alkaline conditions. GCSC-treated *E. coli* has a more significant photolytic effect on anionic superoxide radical ($O_{2}^{•-}$) formation under VLI, as more $O_{2}^{•-}$ is formed within *E. coli* if the GCSC-treated samples are subjected to VLI. The $O_{2}^{•-}$ exhibits a greater effect in a solution of GCSC than that shown by SC alone under VLI treatment. Gold (III) ions in a GCSC system appear to act as an oxidant by facilitating the electron transfer from citrate under VLI and the formation of AuNPs and $O_{2}^{•-}$ via GCSC photolysis under alkaline conditions. As such, the photolysis of GCSC under VLI is a useful process that can be applied to aPDI.

## 1. Introduction

Citric acid functions as a tridentate ligand, and it is frequently incorporated into beverages to enhance the flavor profile. Citrate, which is derived from citric acid and has mild alkaline properties, is a regulator of acidity in foodstuffs. Citrate exhibits limited reductive capability and is inert toward surrounding molecules, but it is susceptible to oxidation by oxidizing agents, such as reactive oxygen species (ROS), because it has a relatively high oxidation potential [1].

The distinctive physicochemical attributes that characterize gold nanoparticles (AuNPs) include a substantial surface area relative to volume and a marked tendency for biocompatibility within specific dimensional thresholds. Their nanoscale also increases the catalytic attributes needed for ROS modulation; thus, they exhibit surface plasmon resonance (SPR), and they are used for laser-mediated photothermal intervention in oncological applications [2].

Nanoparticles are a particular category of materials at a nanometric scale (1–100 nm) that can be produced using lipids, polymers, or noble metals [3]. Metallic nanomaterials are a special category of nanostructures that are produced using noble metals such as copper, gold, platinum, and silver. It was reported that copper oxide nanoparticles conjugated to the *Annona muricata* L. plant extract could inhibit breast cancer cell lines by triggering apoptosis [4]. Additionally, zinc oxide nanoparticles containing Au prepared via laser ablation and then capped with curcumin nanoparticles (Cur-Au@ZnO NPs) showed activity against the α-haemolysin toxin secreted by *S. aureus* [5].

The synthesis of AuNPs is primarily conducted using reducing agents, such as sodium citrate or tetrahydroborate [6]. Based on chemical reduction, the protocol of Turkevich synthesis is widely applied to generate nanoparticles of metals, particularly gold and silver. In a heated aqueous environment, citrate ions act as reducing and stabilizing agents [1]. Jin et al. proposed a photoinduced method to produce significant quantities of silver nanoparticles (AgNPs) by blending silver nitrate with NaBH_4_ and citrate ions before exposure to fluorescent light at 40 W for 40–70 h [7]. Yang et al. noted that under strong blue light at 1 KW/m^2^ for 90 min (540 J/cm^2^), silver nitrate or gold ions can be reduced by sodium citrate, implying that blue light illumination (BLI) activates citrate ions, enhances the reduction of silver or gold ions, and initiates the formation of AgNPs or AuNPs via a photo-assisted process [8,9].

Gharib et al. showed that the increased citrate reduction potential can be attributed to the H_−1_Citrate• (citrate radicals) produced via hydrogen abstraction by ROS prior to the transfer of a single electron from H_−1_Citrate• to reduce silver or gold ions. Under γ-irradiation radiolysis, this process initiates nucleation and then the growth of AgNPs or AuNPs when citrate is mixed with silver or gold ions [1].

*E. coli*, a gram-negative bacterium, is used in the environment and can be found in the digestive systems of animals. It also acts as a microorganism index that shows the presence of its pathogenic counterparts in test environments. Strains such as *E. coli* O157 are significant pathogens in humans that produce an array of toxins that are linked to various syndromes, including gastroenteritis, meningitis, septicemia, and urinary tract infections [10,11].

The process of antibacterial photodynamic inactivation (aPDI) leads to cell death, which is primarily caused by ROS [12,13]. ROS are either reactive oxygen-containing molecules or free radical species, such as hydroxyl radicals (•OH), $O_{2}^{•-}$, peroxyl radicals (ROO•), and singlet oxygen (^1^O_2_). After a photosensitizer is activated to its excited state, an $O_{2}^{•-}$ or ^1^O_2_ species is formed via photolysis [14,15]. Previously, *E. coli*, *S. aureus*, and methicillin-resistant *S. aureus* (MRSA) were inactivated by DNA cleavage triggered by the production of $O_{2}^{•-}$ via blue or violet illumination for riboflavin or riboflavin-5′-phosphate (FMN) [15]. FMN is promoted to the electronically excited state via photolysis and the production of the reactive superoxide radical $O_{2}^{•-}$. It was also reported that when catechin was illuminated by BLI under alkaline conditions, $O_{2}^{•-}$ was formed by photosensitized oxidation, thereby increasing the deactivation of *Acinetobacter baumannii*, including a carbapenem-resistant *Acinetobacter baumannii*. This detection via the photo-oxidation of catechin provided a secure way to deactivate environmental microbes [16]. With tetracycline (TC) illumination by BLI at a pH of 7.8, $O_{2}^{•-}$ was produced via TC photolysis, which increased the deactivation of *E. coli* and multidrug-resistant (MDR) *E. coli*. A 96.6% deactivation rate of MDR *E. coli* was attained, and MDR *E. coli* viability was inhibited by 4 to 5 logs with TC under BLI. The finding regarding TC under BLI thus provides a novel method to deactivate MDR *E. coli* [17]. Therefore, aPDI is an effective strategy to overcome bacterial resistance because it operates independently of the specific bacterial resistance mechanism [18].

It has been reported that UV and X-ray mediate ROS generation, which occurs due to AuNPs. Smaller-diameter AuNPs have a larger surface; thus, more ROS are produced [19]. Vankayala et al. observed the photosensitization of ^1^O_2_ via the irradiation of metal nanoparticles, including AuNPs, using a 0.1 KW mercury lamp [20]. Pasparakis showed that the synthesis of citrate-stabilized AuNPs led to an SPR peak at 524 nm, which was attributable to the generation of ^1^O_2_ under light activation. Subsequent irradiation with a nanosecond laser source at 532 nm greatly increased the ^1^O_2_ production; thus, the light-induced generation of ^1^O_2_ was attributed to direct photosensitization by AuNPs [2]. Chadwickc et al. reported that if citrate-stabilized nanoparticles were irradiated using a continuous-wave diode-pumped solid-state laser operating at 532 nm and 1000 mW, a high level of ^1^O_2_ photogeneration was observed_,_ which was facilitated by AuNPs [21].

The antimicrobial potential of AuNPs is the subject of more than seventy studies due to their distinctive physicochemical attributes [22]. Around eight studies have demonstrated either negligible or very weak antibacterial properties for AuNPs, and the remainder exhibit varying degrees of antibacterial activity [23]. The toxicity of AuNPs is intricate due to the presence of co-existing compounds such as citrate and gold (III) ions during photomutagenicity testing [24]. Wang et al. showed that the AuNPs that are produced in a solution using citrate reduction methods are non-toxic and non-mutagenic, but they are photo-mutagenic toward bacteria. This photomutagenicity is attributable to the presence of citrate and gold (III) ions formed via light irradiation for 15 min using a 300 W Xe lamp rather than the gold nanoparticle itself [24]. The photomutagenicity is due to both citrate and gold (III) ions generating free radicals that cause DNA damage and mutations [24]. In our previous study, VLI was found to be a novel way to carry out the photolysis of SC upon the addition of GC (GCSC) [25]. After VLI treatment on GCSC at 60 W/m^2^ for 90 min, the inhibition rate of WiDr colon cancer cells reached 80.2%, and spherical AuNPs with a mean diameter of 6 nm could be formed via GCSC under VLI, mainly owing to the ROS formed via GCSC photolysis under alkaline conditions. It would be of interest to examine whether GCSC-treated bacteria that are exposed to visible light could be inhibited by ROS production through a charge transfer process using photosensitizers.

Citrate-stabilized nanoparticles support the formation of ROS, albeit with a low quantum yield [21]. However, there is limited knowledge regarding the influence of colored light on photolytic processes that involve GCSC for AuNP and ROS formation. This study aims to investigate how the photolysis of SC and GCSC increases ROS generation and inhibits microbial viability at a faster rate. It also explores the impact of different colored light on AuNP production and the effect of aPDI on *E. coli* using AuNPs generated via GCSC under light illumination. Furthermore, this study demonstrates the formation of ROS using photo-activated SC and GCSC photolysis under visible light. The effect of GCSC under the illumination of visible light on bacterial viability is used as an index to determine the technique’s effectiveness.

## 2. Methods and Materials

### 2.1. Reagents

Gold (III) chloride trihydrate was purchased from Alfa Aesar (Ward Hill, MA, USA). Anhydrous sodium citrate was provided by Acros Organics (Morris Plains, NJ, USA). Suspensions of AuNPs with a diameter of 30 nm (Cat. # 753629), without stabilization in citrate buffer, were bought from Sigma-Aldrich Co. (St. Louis, MO, USA). Nitrotetrazolium blue chloride (NBT) was purchased from Bio Basic Inc. (Markham, ON, Canada). Deionized water was generated using the Milli-Q water purification system (Merck KGaA, Darmstadt, Germany) and was used for the preparation of all the solutions used in this study. Fourfold-diluted AuNP solutions (D--AuNPs) from a bare AuNP suspension were produced in a 0.1 mM PBS solution to give solutions containing about 4.5 × 10^10^ particles/mL.

### 2.2. Photolytic Reaction Setup

**Figure 1:**
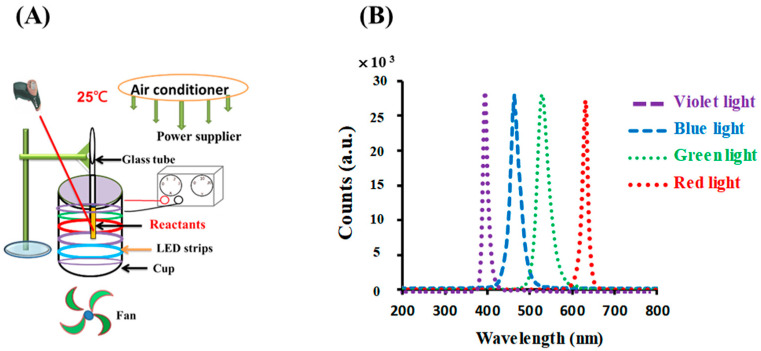
(**A**) Photolytic system and (**B**) emission spectra for LED lamps used in this study.

The photolytic reaction setup consisted of an illumination setup, including opaque plastic components with a light source, as detailed in previous studies [18,26]. For the photoreaction, six light-emitting diodes (LED) (30 cm, DC 12 V 5050, vitaLED Tech. Co., Tainan, Taiwan) were fitted to the inner side of an opaque plastic cup (height of 8 cm and diameter of 7 cm), as shown in Figure 1A. The intensity of the colored light was kept at 10 W/m^2^, as measured using a solar power meter (TM-207, Tenmars Electronics, Taipei, Taiwan). For the photolysis experiments, the temperature was kept at 25 ± 3 °C using an air conditioner equipped with an electric fan and was measured using an infrared thermometer (MT4, Raytek Co, Santa Cruz, CA, USA). This arrangement created an environment for the photoreaction. The reaction solution, 0.1 mM gold (III) chloride trihydrate upon the addition of 4.53 mM sodium citrate, was placed in test tubes on the upper rim of the cup.

As shown in Figure 1B, the LED lamps emitted blue, green, red, and violet light with peak wavelengths at 448, 529, 632, and 403 nm, respectively; their spectral widths at half height were 25, 31, 14, and 16 nm, respectively, as measured using a UV-vis miniature fiber-optic spectrometer (RB1020, Rainbow Light Tech. Co., Taoyuan, Taiwan).

### 2.3. Influence of Colored Light on the Development of AuNPs

Sodium citrate (SC) acts as a weak reductant. In our previous study, there was much more electron transformation during SC photolysis under VLI than that under BLI. In addition, an electron-rich environment increases the amount of $O_{2}^{•-}$ generation after SC under VLI treatment [25]. The effects of the photolysis of SC on the formation of AuNPs upon the addition of gold chloride utilizing colored light are inspected in this study.

In the photosensitized reaction, colored light treatments were used to illuminate the sample solutions. The experiment was conducted at pH 7.2 to determine the effect of citrate on gold (III) ions following exposure to colored light. A solution containing 4.53 mM of sodium citrate was mixed with 0.1 mM of gold (III) chloride in H_2_O. One milliliter of this mixture solution was placed in a test tube on the upper rim of the photolytic system and subjected to colored light illumination at an intensity of 10 W/m^2^ for 60 min or left in the dark as a control. The temperature was kept at 25 ± 3 °C using an air conditioner and an electric fan. A UV-vis spectrophotometer (U-2900, Hitachi, Tokyo, Japan) was utilized to measure the SPR band of the AuNPs. Absorbances from 200 to 800 nm were collected.

The particle size of the AuNPs was measured utilizing a Joel JEM-2100 transmission electron microscope (TEM) (JOEL Ltd., Tokyo, Japan) at 200 KV to acquire TEM images of the samples. Before the TEM examination, AuNPs in aliquots were dripped onto a carbon-coated copper grid and dried at 30 °C in an oven for two days.

### 2.4. Effect of GC, SC, GCSC, and D—AuNPs on E. coli under VLI

The deactivation of *E. coli* under VLI treatment was examined using GC (0.1 mM gold (III) chloride trihydrate in 100 mM potassium phosphate buffer at pH 7.8, i.e., PBS), SC (4.53 mM sodium citrate in PBS), GCSC (the GC solution to which 4.53 mM sodium citrate in PBS was added), and diluted AuNPs solutions (D--AuNPs) in a photolysis system.

*E. coli* of the DH5α strain (NCBI Taxonomy ID: 668369) was cultured in LB broth at 37 °C overnight. When the bacteria reached an optical density at 600 nm (OD_600_) of 0.5 (approximately 7.6 × 10^6^ CFU/mL), 1.0 mL of *E. coli* culture was transferred to a 1.5 mL centrifuge tube. After centrifuging for 10 min at 13,000 rpm, the supernatant was removed and a 1 mL solution of GC (0.1 mM), SC (4.53 mM), GCSC, or D--AuNPs was added to the bacterial suspension. These mixtures were then placed in glass tubes and subjected to VLI or kept in the dark. The experiments were labeled as (A) bacteria treated with PBS left in the dark as a control; (B) bacteria treated with PBS and subjected to VLI at 10 W/m^2^ for 60 min; (C) bacteria treated with GC, SC, GCSC, and D--AuNPs in PBS and kept in the dark; and (D) bacteria treated with GC, SC, GCSC, and D--AuNPs in PBS and subjected to VLI at 10 W/m^2^ for 60 min.

The plates and tubes for the dark control were covered with thin aluminum foil to avoid light exposure. The photolytic reaction temperature was maintained at 25 ± 3 °C. After the photoreaction, 0.2 mL of the bacterial solution was transferred onto Luria agar plates and incubated at 37 °C overnight. The survival of *E. coli* was measured using viable plate colony counts (CFUs). The extent of the bacterial inactivation was quantified using the log reduction formula [=log (D/V)], where V is the number of CFUs after VLI [V], and D is the control CFUs in the dark [D].

### 2.5. Effects of GCSC on E. coli under Colored Light Illumination

This investigation determined the effect of blue, green, red, and violet light illumination on the deactivation of *E. coli* using GCSC in a photolysis system. The *E. coli* sample was produced using the method described in Section 2.4. After centrifuging for 10 min at 13,000 rpm, the supernatant was removed and 1 mL solutions of GCSC were added to the bacterial suspension. These mixtures were then placed in glass tubes and subjected to either blue, green, red, or violet light illumination at an intensity of 10 W/m^2^ or kept in the dark. The survival of *E. coli* was measured using CFUs using a method similar to that given in Section 2.4.

### 2.6. Observation of $O_{2}^{•-}$

$O_{2}^{•-}$ is produced during a redox reaction. These free radical species are investigated utilizing direct and indirect methods. Direct investigation uses specialized apparatus such as an electron paramagnetic resonance (EPR) spectrometer, and the indirect methods use a nitro blue tetrazolium (NBT) reduction assay for biochemical analysis [14]. NBT acts as a scavenger that shows the existence of $O_{2}^{•-}$ and the degree of NBT reduction expressing the presence of $O_{2}^{•-}$ and is used to quantify the $O_{2}^{•-}$ level [14,27].

To determine the effect of GCSC under VLI on $\;O_{2}^{•-}$ formation, an NBT reduction analysis was adapted from the technique of previous studies [14]. All the necessary reagents were prepared prior to each photoreaction. Three scenarios were considered: (A) a solution containing 0.1 mM GC and 0.12 mM NBT in PBS; (B) a solution containing 4.53 mM SC and 0.12 mM NBT in PBS; and (C) a solution containing 0.1 mM GC, 4.53 mM SC, and 0.12 mM NBT in PBS at pH 7.8. These solutions were subjected to VLI at an intensity of 10 W/m^2^ for 0–30 min. During a photolytic reaction, $O_{2}^{•-}$ is generated, which reduces NBT to produce formazan via an electron transformation. Formazan is detectable at 560 nm.

### 2.7. $O_{2}^{•-}$ Detection Using GCSC-Treated E. coli Subjected to BLI or VLI

$O_{2}^{•-}$ detection in *E. coli* when the GCSC-treated *E. coli* is exposed to either BLI or VLI was measured utilizing the NBT reduction method. This method follows established protocols with slight adjustments [26]. The *E. coli* sample was produced using the method given in Section 2.4, but a 1.0 mL *E. coli* culture was placed in a microcentrifuge tube and diluted to an OD_600_ of 0.05 (about 7.6 × 10^5^ CFU/mL). After centrifuging for 10 min at 13,000 rpm, the supernatant was removed, followed by the addition of a 1 mL reaction solution. All the chemicals were freshly prepared prior to each test.

The concentrations of GC, SC, and NBT in the solution were adjusted to 0.1 mM, 4.53 mM, and 1.2 mM, respectively. The mixture was transferred to a glass tube and subjected to either BLI or VLI at an intensity of 10 W/m^2^ or was kept in the dark for 60 min. After illumination, the mixture was centrifuged for 10 min at 13,000 rpm, and the supernatant was then removed. To extract the reduced NBT, 1 mL of dimethyl sulfoxide (DMSO) was added, and the mixture was allowed to incubate for 30 min. As a result of the photolytic reaction, $O_{2}^{•-}$ reduced NBT to form blue formazan, which was measured at 560 nm.

### 2.8. Statistics

Each experiment was conducted on consecutive days and was repeated three times. For each independent test, the *E. coli* samples were treated under specific testing conditions. The results were presented as mean ± standard deviation (SD) for a minimum number of these tests. A homoscedastic two-sample *t*-test was used to identify differences between the population means. A *p* value < 0.05 indicates statistical significance.

## 3. Results and Discussion

### 3.1. The Alternation of Citrate Mixed with Gold (III) Ions under Colored Light Illumination

**Figure 2:**
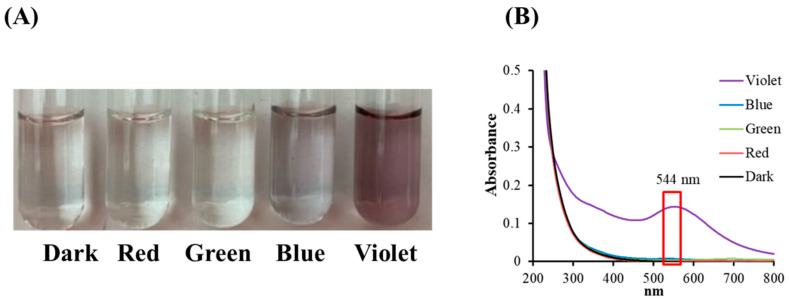
(**A**) Color changes and (**B**) absorbance spectra for sodium citrate mixed with gold (III) chloride under blue, green, red, and violet light illumination at 10 W/m^2^ for 60 min.

The effects of colored light illumination on citrate mixed with gold (III) ions were determined by analyzing the color, or spectral alternation, in the reaction solutions following light illumination. Figure 2A shows that the color changes are insignificant for illumination by blue, green, and red light, but that under VLI, the mixed solution obtains a purplish color. This shows that the gold (III) ions in the citrate solution undergo transformation through a photochemical reaction.

Figure 2B shows the spectra for citrate mixed with gold (III) ions under colored light illumination. The spectral data in Figure 2B show a single signal with a surface plasmon resonance (SPR) peak at 544 nm during VLI, but illumination with blue, green, or red light produces no significant change. Figure 2 shows that there is no significant color or spectral changes in citrate mixed with gold (III) ions under illumination by blue, green, or red light, but exposure to violet light at 10 W/m^2^ for 60 min (3.6 J/cm^2^) changes its properties.

**Figure 3:**
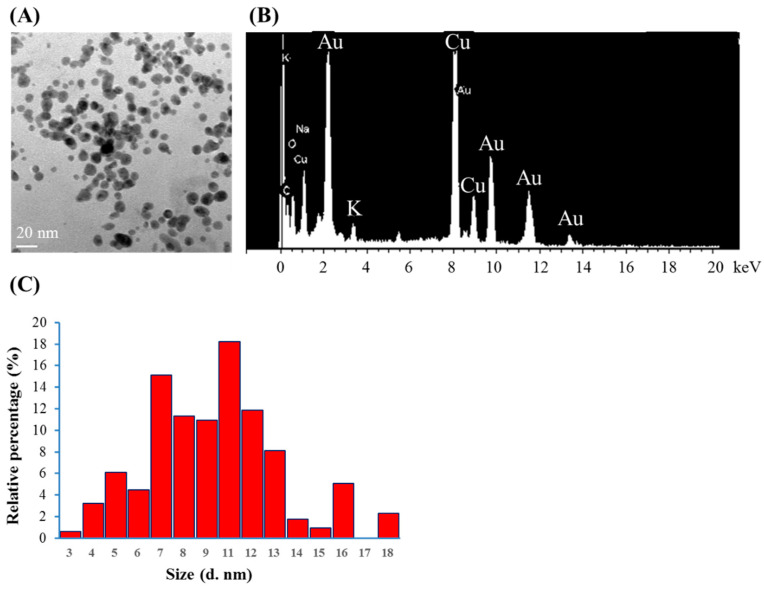
(**A**) TEM images of AuNPs, (**B**) spot EDS analysis indicating the presence of gold, and (**C**) the diameters of AuNPs produced from citrate mixed with gold (III) ions under VLI at 10 W/cm^2^ for 60 min, as calculated using a TEM image.

Citrate mixed with gold (III) ions treated with VLI produces expressed AuNPs, as shown in Figure 3. The nanoparticles were measured using TEM. Figure 3A shows that more than 79% of the AuNPs are spherical for citrate mixed with gold (III) ions under VLI at 10 W/m^2^ for 60 min. Figure 3B shows that many AuNPs have a diameter of 3 to 18 nm, with an average value of 9 nm.

It has been reported that GC can be reduced by SC under intense BLI (1 kW/m^2^ for 1.5 h (540 J/cm^2^)) to form AuNPs [8,9]. As shown in Figure 2 and Figure 3, AuNPs can be formed via citrate mixed with gold (III) ions and treated with VLI at 10 W/m^2^ for 60 min (3.6 J/cm^2^), whereas there are no significant color or spectral changes observed with blue, green, or red light illumination under the same conditions. This indicates that the photolysis efficiency of citrate when mixed with gold (III) ions is primarily regulated by the light quality, with violet light being the most powerful among all the light sources examined in this study.

### 3.2. $O_{2}^{•-}$ Detection in SC and GCSC under VLI Treatment

**Figure 4:**
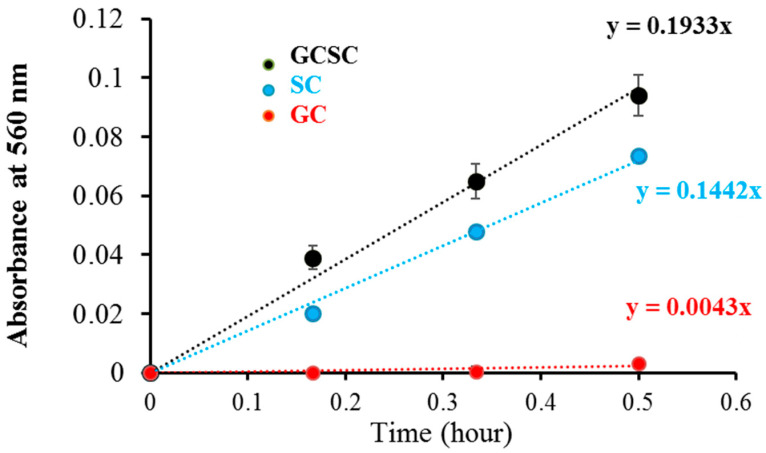
The photolysis effect of GC, SC, and GCSC on NBT reduction, as $O_{2}^{•-}$ is produced from GC, GCSC, and SC treated with NBT and exposed to VLI at 10 W/m^2^ for 0–30 min.

$O_{2}^{•-}$ detection in GC, SC, and GCSC under VLI was measured utilizing the NBT reduction technique under alkaline conditions. The effects of GC, SC, and GCSC under VLI at 10 W/m^2^ for 0–30 min on NBT reduction, which are a measure of $O_{2}^{•-}$ generation, are presented in Figure 4, which shows that the effects of SC and GCSC photolysis on NBT reduction increase over a 30 min period under VLI. The respective rates of $O_{2}^{•-}$ production due to GC, SC, and GCSC under VLI are 0.0043, 0.1442, and 0.1933 (h^−1^), as shown in Figure 4; thus, SC and GCSC are photosensitizers that are sensitive to violet light. The reduction gradient for GC indicates minimal production of these radicals, but the reduction gradient for GCSC under VLI at an intensity of 10 W/m^2^ for 60 min is significantly steeper, indicating that the photolysis of GCSC under VLI leads to a significant increase in the creation of ${\;O}_{2}^{•-}$ via electron transfer.

Figure 4 shows that the NBT reduction rates of the ${\;O}_{2}^{•-}\;$ generation of the VLI-treated SC and GCSC are 0.1442 and 0.1933 (h^−1^), respectively. The redox potential of (Acetone-1,3-dicarboxylate + CO_2_)/H_−1_Citrate• is less than −1.2 V_NHE_ [1]. The redox potential of GC (Au${\mathrm{Cl}}_{4}^{-}$) is 930 mV. GCSC is more responsive under violet light irradiation. Gold (III) ions in a GCSC system act as an oxidant; thus, electron transfer from SC is increased under VLI, which promotes the generation of $O_{2}^{•-}$ via GCSC under VLI [25].

The Turkevich method is used to produce AgNPs and AuNPs. Citrate ions act as both reducing agents and stabilizers in heated aqueous solution [1]. Al Gharib et al. demonstrated that under γ-irradiation radiolysis, the reducing power of citrate is enhanced; thus, AgNPs or AuNPs form more efficiently from silver or gold ions mixed with citrate [1]. In Figure 4, the increasing gradient for NBT reduction indicates that under VLI, the SC solution generates an increasing amount of $O_{2}^{•-}$ in a time-dependent manner, which implies that the $O_{2}^{•-}$ formed in SC under VLI results from an electron transfer process.

Under alkaline conditions (pH > 7.4), citrate dissociates all the protons on its three carboxyl groups and, through the scavenging of $O_{2}^{•-}$ and hydrogen atom abstraction, forms a carbon-centered radical at its methylene group, i.e., (^−^O_2_CC^•^H)(^−^O_2_CCOH)(H_2_CCO_2_^−^) or H_−1_Citrate• [1]. The free radical species, H_−1_Citrate•, formed under γ-radiolysis is a much stronger reductant compared to citrate and undergoes decarboxylation to form acetone-1,3-dicarboxylate [1].

This study suggests equations for the photoreaction of citrate at alkaline pH: Equations (1)–(3):

(1) $$({\;\;}^{-}O_{2}{\mathrm{CCH}}_{2})({\;\;}^{-}O_{2}\mathrm{CCOH})(H_{2}{{\mathrm{CCO}}_{2}}^{-})\;\overset{\;hv\;}{\to }({\;\;}^{-}O_{2}{\mathrm{CC}}^{•}H)({\;\;}^{-}O_{2}\mathrm{CCOH})(H_{2}{{\mathrm{CCO}}_{2}}^{-})\;+\;H•\;\mathrm{Citrate}\;\;\;\;\;H_{-1}\mathrm{Citrate}•$$

$$({\;\;}^{-}O_{2}{\mathrm{CC}}^{\text{•}}H)({\;\;}^{-}O_{2}\mathrm{CCOH})(H_{2}{{\mathrm{CCO}}_{2}}^{-})+({\;\;}^{-}O_{2}{\mathrm{CC}}^{\text{•}}H)({\;\;}^{-}O_{2}\mathrm{CCOH})(H_{2}{{\mathrm{CCO}}_{2}}^{-})\;\overset{\;hv\;}{\to }\;H_{-1}\mathrm{Citrate}•\;H_{-1}\mathrm{Citrate}•$$

(^−^O_2_CCH_2_)(^−^O_2_CCOH)(H_2_CCO_2_^−^) + (^−^O_2_CCH_2_)C=O(H_2_CCO_2_^−^) + CO_2_ + H^+^ + 2e^−^
 Citrate        Acetone-1,3-dicarboxylate(2)

Overall,

(3) $$2\;({\;\;}^{-}O_{2}{\mathrm{CCH}}_{2})({\;\;}^{-}O_{2}\mathrm{CCOH})(H_{2}{{\mathrm{CCO}}_{2}}^{-})\;\overset{\;hv\;}{\to }({\;\;}^{-}O_{2}{\mathrm{CCH}}_{2})({\;\;}^{-}O_{2}\mathrm{CCOH})(H_{2}{{\mathrm{CCO}}_{2}}^{-})\;2\;\mathrm{Citrate}\;\mathrm{Citrate}\;({\;\;}^{-}O_{2}{\mathrm{CCH}}_{2})C=O(H_{2}{{\mathrm{CCO}}_{2}}^{-})+{\mathrm{CO}}_{2}+H^{+}+2e^{-}\;\text{Acetone-1,3-dicarboxylate}$$

In Equation (1), the radical species, H_−1_Citrate•, is generated via hydrogen atom abstraction from citrate subjected to light illumination. Apparently, decarboxylation occurs during the oxidation of H_−1_Citrate•, as evidenced by the formation of the products acetone-1,3-dicarboxylate and CO_2_ [1,25].

The oxidation of citrate results in the formation of acetone dicarboxylate (DC^2−^) that acts as a reducing agent during the formation AuNPs [28]. A rise in the hydroxide ions results from the decomposition of DC^2−^ [29], as shown in Equation (4).

The decomposition products, e.g., acetone, reduce trivalent gold (Au^3+^) and lead to a complete conversion to Au^0^. Therefore, DC^2−^ acts as an auxiliary reducing agent [6] (shown in Equation (5)). During the synthesis of AuNPs using SC as a reducing agent, the Turkevich mechanism consists of two consecutive reducing steps, i.e., Au^3+^ → Au^+^ → Au^0^, with the second step being the rate-determining step [29].

(4) $$({\;\;}^{-}O_{2}{\mathrm{CCH}}_{2})C=O(H_{2}{{\mathrm{CCO}}_{2}}^{-})+2\;H_{2}O\;\overset{\;}{\to }C_{3}H_{6}O+2\;{\mathrm{OH}}^{-}+2\;{\mathrm{CO}}_{2}$$

Acetone-1,3-dicarboxylate

(5) $$2\;{{\mathrm{AuCl}}_{4}}^{-}+6\;H_{2}O+3\;C_{3}H_{6}O\overset{\;}{\to }4\;{\mathrm{Au}}^{0}+12\;H^{+}+12\;{\mathrm{Cl}}^{-}+9\;H_{2}O$$

The molar ratio between SC and HAuCl_4_^−^ has a profound effect on the particle size of AuNPs formed in the reaction solutions [30]. Smaller AuNPs can be obtained by increasing the molar ratio of SC vs. HAuCl_4_^−^ owing to higher nucleation and consequently higher concentrations of generated nuclei [31,32,33]. Moreover, acetone dicarboxylate (DC^2−^) formed as an intermediate of citrate oxidation plays a key role as a stabilizer and a reducing agent that is stronger compared to SC in the reaction between SC and HAuCl_4_^−^ of a molar ratio (in mM) of 2.5:0.25 [29]. The formation of DC^2−^ via decarboxylation of SC can be strongly enhanced by photolysis in Fe(III) citrate/citric acid [34] as the hydroxyl group adjacent to the central carboxyl group facilitates the process [35]. In the current study, the resultant gold species is most likely Au^0^, given the high molar ratio (in mM) of 4.5:0.10 and the powerful reducing agent, DC^2−^, which can form during the photolysis of SC. Nevertheless, the parameters of the predominant gold species currently examined in this study need to be further explored in future studies using techniques such as X-ray photoelectron spectroscopy (XPS), which determines the oxidation states. In addition, the differences in the crystal lattice parameters, e.g., shape, type, and size, between AuNPs and Au(OH)_3_ when crystallized can be differentiated according to their 2θ values using X-ray diffraction (XRD).

### 3.3. Effect of GCSC Exposed to Colored Light on E. coli Survival

**Figure 5:**
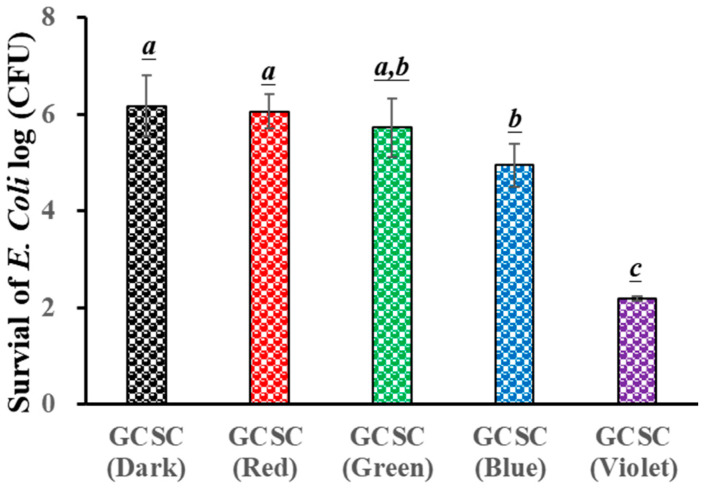
Effect of GCSC under blue, green, red, and violet light illumination at 10 W/m^2^ for 60 min on the viability of *E. coli*. The letters above each bar show a statistically significant difference between the means of two groups when *p* < 0.05.

The influence of GCSC exposure to colored light on the survival of *E. coli* is evaluated. Figure 5 reveals that there is little significant difference in the survival rate of *E. coli* under blue, green, and red light illumination for 60 min when treated with GCSC in the absence of light. For GCSC exposed to VLI at 10 W/m^2^ for 60 min, a significant increase is noticed in the deactivation rate of *E. coli*. GCSC that is subjected to VLI better deactivated *E. coli* under alkaline conditions, as shown in Figure 5. This shows that, in terms of the inactivation rate, the photolysis efficiency of citrate mixed with gold (III) ions is primarily regulated by light quality, and violet light is the most sensitive among the light sources examined in this study in terms of suppressing pathogenic bacteria.

It was reported that FMN, after exposure to VLI, more effectively inactivated *S. aureus*, while a similar finding was observed for *S. aureus* after long-term exposure to BLI under the same circumstances [36]. Using a proper photosensitizer such as FMN, low-energy VLI can inhibit pathogenic bacteria. The effects of GCSC exposed to VLI and BLI on the survival of *E. coli* are 2.2 and 4.95 log CFU/mL, respectively, as shown in Figure 5. A previous study revealed that when the GCSC was exposed to BLI and VLI at 2.0 mW/cm^2^ for 1.5 h, the respective survival rates of WiDr cells were 95.9 and 42.3% [25]. At low-energy doses, GCSC exposed to VLI exhibited an increased photodynamic therapy effect [25].

### 3.4. Effects of GC, SC, GCSC, and D--AuNPs on E. coli Survival When Subjected to VLI

**Figure 6:**
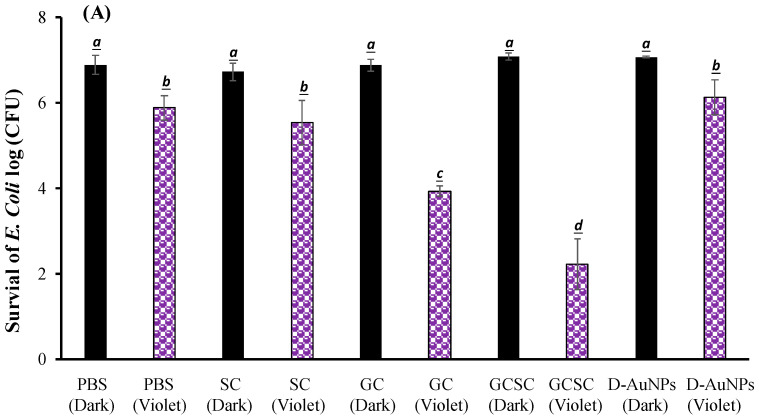

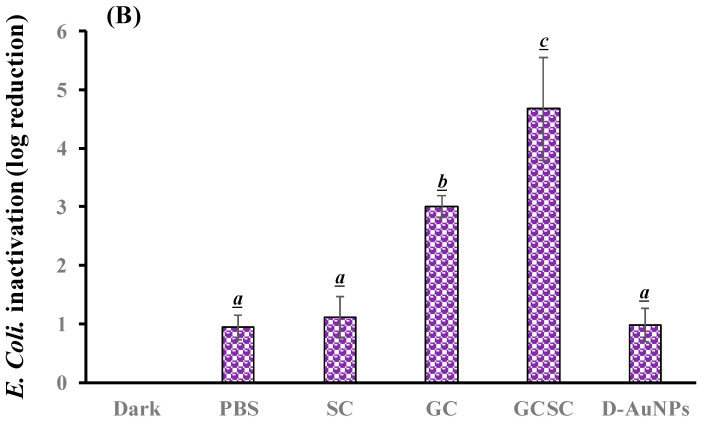
Effect of SC, GC, GCSC, and D--AuNPs under VLI at 10 W/m^2^ for 60 min on the (**A**) viability and (**B**) inactivation rate of *E. coli*. The letters above each bar show a statistically significant difference between the means of two groups when *p* < 0.05.

The effects of GC, SC, GCSC, and D--AuNPs on *E. coli* survival when subjected to violet light were determined under alkaline conditions. In Figure 6A, the bacteria were treated with PBS and left in the dark; they exhibited approximately 7.6 × 10^6^ CFU/mL (indicated as 6.8 log CFU/mL). Figure 6A reveals that there is no significant difference in the survival rate of *E. coli* with or without treatment of GC, SC, GCSC, and D--AuNPs for 60 min in the dark under alkaline conditions. There is a significant difference in the survival rate of *E. coli* treated with VLI. As shown in Figure 6B, when exposed to VLI at 10 W/m^2^ for 60 min, the inhibition of *E. coli* increases by 0.94 log compared to the control (dark) treatment. Disinfection using 405 nm light on both gram-positive and gram-negative bacteria has been reported to reduce bacterial counts [37]. The inactivation of bacteria by 405 nm VLI is caused by the intracellularly formed ROS [37]; this is primarily due to the endogenous porphyrins acting as photosensitizers [38]. Light with a wavelength of more than 430 nm does not eliminate *S. aureus* cells [38].

For *E. coli* treated with SC, GC, GCSC, and D--AuNPs under VLI at 10 W/m^2^ for 60 min, there is a significant difference in the deactivation rate compared to *E. coli* in the dark treatment. The average inhibitory effects of SC, GC, GCSC, and D--AuNPs on the VLI-induced *E. coli* inhibition are 1.12, 2.95, 4.67, and 0.98 logs, respectively, under alkaline conditions (Figure 6B).

For *E. coli* treated with GC under VLI, there is a significant increase in the inactivation rate of *E. coli* compared to that which occurs in the dark treatment. This study shows that if *E. coli* is exposed to VLI at an intensity of 10 W/m^2^ for 60 min and treated with GC, there is a significant decrease in bacterial activity, with the inactivation rate reaching 2.95 logs, as shown in Figure 6B. Proteins such as the mitochondrial enzyme cytochrome c oxidase, a component of mitochondrial complex IV, strongly absorb violet light, particularly within the wavelength range of 400–410 nm [25,39]. Mitochondria, which are rich in photoreceptors, also absorb visible light. Citrate, which is a key metabolite that is produced in the mitochondria and is involved in the tricarboxylic acid cycle (TCA cycle), is transported into the cytoplasm through the mitochondrial citrate carrier [40]. When *E. coli* is exposed to VLI in the presence of GC, intracellular photosensitizers, including citrate, are particularly susceptible to violet light, with $O_{2}^{•-}$ being formed due to these photosensitizers. The presence of GC under VLI increases the occurrence of $O_{2}^{•-}$ through an electron transfer process by intracellular endogenous photosensitizers.

For *E. coli* treated with GC and GCSC under VLI, there is a significant difference in the deactivation rate of *E. coli* in these treatments. The average suppressive effects of GC and GCSC due to the VLI-induced inhibition of *E. coli* under alkaline conditions are 2.95 and 4.67 logs, respectively (Figure 6B). When GC is exposed to VLI and endogenous photosensitizers are used, there is a smaller reduction in *E. coli* viability than for an exogenous photosensitizer such as GCSC. If GC is added, the exposure of GCSC to violet light decreases the viability of *E. coli* to a much greater extent than that which occurs when only endogenous photosensitizers are the species present intracellularly.

AuNPs induce cell death directly through photolysis without a photosensitizer [21]. Studies have shown that UV irradiation increases the production of ROS in citrate-stabilized AuNPs and that the catalytic effect of photolysis increases ROS yield [19]. This study produced D--AuNPs using fourfold dilutions of bare AuNP suspensions (30 nm diameter) in 0.1 mM PBS without citrate buffer being added. There is no significant difference (*p* = 0.16) in the survival rate of *E. coli* treated with or without D--AuNPs under VLI. The inhibitory effect of D--AuNPs under VLI results in the suppression of *E. coli* by 0.98 log, as shown in Figure 6B. This inhibitory effect is attributed to violet light illumination and is facilitated by endogenous photosensitizers. Cheng et al. also noted that WiDr cancer cells treated with D--AuNPs do not exhibit a decrease in percentage under dark conditions or under BLI or VLI at 20 W/m^2^ for 90 min under alkaline conditions [25]. Wang et al. used the citrate reduction method to demonstrate that AuNPs synthesized in an aqueous solution have no mutagenicity or toxicity toward cells but instead exhibit a photomutagenic effect on bacteria [24]. The bacterial photomutagenicity that is attributable to both citrate and Au^3+^ is likely due to the generation of free radicals from citrate decarboxylation, which is induced by light or by oxidation by gold (III) ions on adjacent molecules [24].

### 3.5. $O_{2}^{•-}$ Detection in GCSC-Treated E. coli Subjected to BLI or VLI

**Figure 7:**
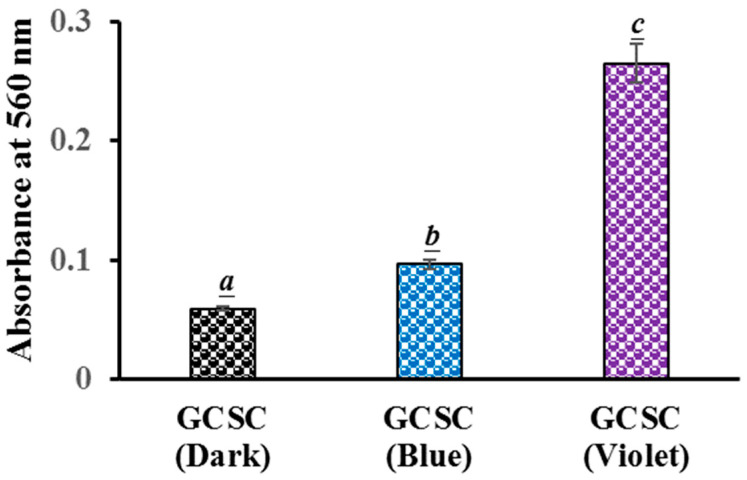
Effect of GCSC-treated *E. coli* subjected to BLI or VLI at 10 W/m^2^ for 60 min on NBT reduction. The letters above each bar show a statistically significant difference between the means of two groups when *p* < 0.05.

The generation of $O_{2}^{•-}$ in GCSC-treated *E. coli* after exposure to BLI or VLI was studied using the NBT reduction method. For *E. coli* treated with GCSC under VLI, there is a significant difference in the level of $O_{2}^{•-}$ generation in *E. coli* compared to that under BLI at 10 W/m^2^ for 60 min. The absorbances at 560 nm, indicating the ROS generation in *E. coli*, were 0.10 and 0.27 for *E. coli* treated with GCSC under BLI and VLI, respectively, as shown in Figure 7. This suggests a significant increase in $O_{2}^{•-}$ formation in *E. coli* after GCSC treatment with VLI. Cheng et al. reported that in ROS generation from GCSC photolysis, GC increased the generation of $O_{2}^{•-}$ via an electron transfer process, leading to the inhibition of WiDr cells. The cancer cells were suppressed by 80.2% after treatment with SCGC under VLI at 6.0 mW/cm^2^ for 1.5 h [25]. Additionally, the presence of ROS was confirmed by propidium iodide (PI) due to the high cell membrane permeability and a concurrent enhancement in the number of PI-positive nuclei within the WiDr cells [25].

**Figure 8:**
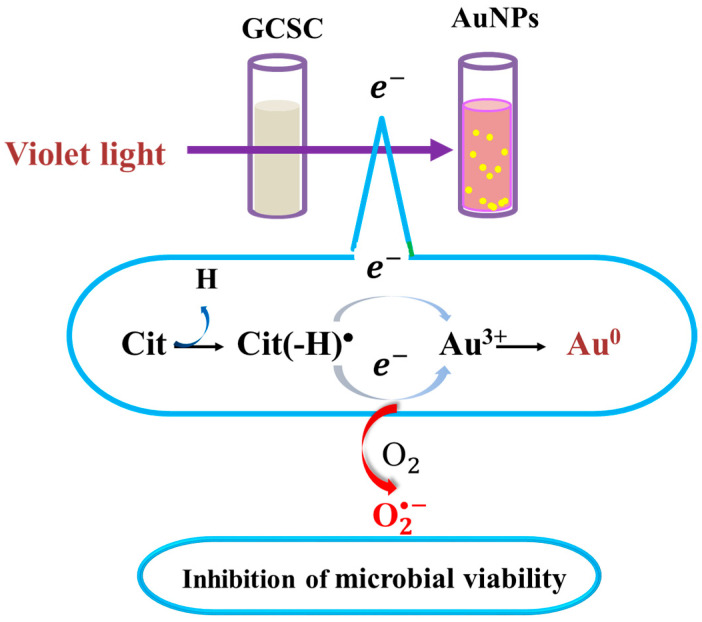
Effects of GCSC on inactivation of bacteria and formation of AuNPs under VLI treatment. The yellow spots represent gold nanoparticles.

There is a significant increase in the photolytic effect of GCSC-treated *E. coli* on the amount of $O_{2}^{•-}$ under VLI, as shown in Figure 7. In Figure 5, *E. coli* is suppressed by GCSC photolysis, which is more effective under VLI compared to blue, green, or red light. Furthermore, the SPR peak at 544 nm, AuNPs, and color change achieved via GCSC under VLI are insignificant under illumination by blue, green, and red light, as shown in Figure 2. *E. coli* is suppressed and the presence of AuNPs is observed when GCSC is under VLI, indicating that the photolysis efficiency of citrate mixed with gold (III) ions is mainly influenced by the light quality. Additionally, gold (III) ions in the citrate solution which undergo transformation through a photochemical reaction via an electron transfer process, as shown in Figure 8.

As shown in Figure 4, GCSC under VLI treatment exhibits higher photosensitivity. The formation of $O_{2}^{•-}$ in GCSC-treated *E. coli* under VLI is significantly increased, as shown in Figure 7. Following VLI, SC and GCSC exhibit an average inhibitory effect on *E. coli* of 1.12 and 4.67 log reductions, respectively. AuNPs are at the nanoscale and show excellent catalytic capabilities for ROS; they have been widely used in laser photothermal cancer therapy [2]. AuNPs exhibit three operational modes when inducing cell death by irradiation: (A) hyperthermia, the rapid conversion of absorbed light energy into heat; (B) nanoparticle-assisted photodynamic therapy, whereby the efficiency of a standard photosensitizer is enhanced by nanoparticles that amplify the plasmon field; and (C) direct photolysis without a photosensitizer [21]. As shown in Figure 4, gold (III) ions in a GCSC system increase the level of $O_{2}^{•-}$ production via GCSC under VLI, which leads to DNA damage and the subsequent mutation of *E. coli*. DNA strand breakage leads to the depletion of intracellular ATP and NAD^+^, resulting in cell death [41]. The photolytic process for GCSC under VLI enhances the production of ROS and the bactericidal inactivation rate. The inhibitory effect of D--AuNPs under VLI results in the suppression of *E. coli* by 0.98 log, as shown in Figure 6B. This inhibitory effect is attributed to violet light illumination and is facilitated by endogenous photosensitizers. Sodium citrate acts as a weak reductant and is sensitive to VLI. Gold (III) ions in a GCSC system act as an oxidant; thus, electron transfer from SC is increased under VLI, which promotes the generation of $O_{2}^{•-}$ via GCSC under VLI. This study demonstrates that the photolysis of GCSC under VLI facilitates the synthesis of AuNPs and significantly inactivates *E. coli*; this is primarily due to the aPDI by induced ROS generated in the violet light–activated GCSC.

Antimicrobial resistance is a primary worldwide health concern for human medicine [42]. The development of new antibiotics often takes decades. High-intensity UVC also belongs in the risk category of UV irradiation due to its high energy [43]. When dealing with visible light or UV irradiation, light of longer wavelength, i.e., less energy, is considered relatively safe to cells. The key advantage of localized aPDI treatment is the fact that it is independent of the bacterium resistance pattern. The aPDI treatment has become a potential alternative or adjuvant in treating skin and soft tissue infections (SSTIs) [44,45], and it is used to reduce nosocomial infections of the skin by multi-resistant bacteria [46]. At low-energy doses, the photolysis of GCSC by VLI deactivates pathogenic bacteria under specific low-intensity conditions. For a bactericidal agent to be considered effective, it must achieve at least a 3-log reduction in bacterial growth [47]. The data demonstrate that the exposure of GCSC to VLI significantly enhances bacterial inactivation, suggesting that the photolysis of GCSC under VLI can be applied as an effective strategy in aPDI processes.

## 4. Conclusions

GCSC undergoes photolysis under VLI to trigger a cascade of photochemical events. This process, which is initiated by electron transfer, leads to the generation of significant levels of ROS. The formation of AuNPs occurs simultaneously with ROS production, which contributes to the enhanced deactivation of pathogenic bacteria under alkaline conditions.

Violet light is crucial for the photoreaction of GCSC. The photolysis of GCSC under VLI results in a reduction rate of 4.67 logs when inactivating *E. coli* and forms ${\;O}_{2}^{•-}$ under alkaline conditions. The results for aPDI on GCSC indicate that this treatment is effective for both subcutaneous and surface skin infections. An optical fiber is utilized to guide a violet light beam to the infected tissues. This study demonstrates that GCSC under VLI enhances bacterial inactivation and can be used to eradicate bacteria in a wound.

## Data Availability

Data will be made available on request.

## Abbreviations

aPDI	antibacterial photodynamic inactivation
AuNPs	gold nanoparticles
CFUs	viable plate colony counts
D--AuNPs	four-fold diluted AuNPs suspensions
*E. coli*	Escherichia coli
GC	0.1 mM gold (III) chloride trihydrate in PBS
GCSC	GC upon addition of the SC
NBT	nitrotetrazolium blue chloride
$O_{2}^{•-}$	anionic superoxide radicals
PBS	0.1 M potassium phosphate buffer at pH 7.8
ROS	reactive oxygen species
SC	4.53 mM sodium citrate in PBS
SPR	surface plasmon resonance
TEM	transmission electron microscopy
VLI	violet light illumination in phosphate buffered solution at pH 7.8
